# Genomics Detects Japanese and Pacific Sardine (*Sardinops* spp.) Hybrids in the Northeast Pacific Ocean

**DOI:** 10.1002/ece3.74198

**Published:** 2026-09-14

**Authors:** Gary C. Longo, Flora Coden, Miasara Andrew, Kelsey James, Kristen Hinton, Jennifer Topping, Matthew Craig

**Affiliations:** ^1^ Contractor to Ocean Associates, Inc. Southwest Fisheries Science Center La Jolla California USA; ^2^ NOAA National Marine Fisheries Service, Southwest Fisheries Science Center La Jolla California USA; ^3^ Washington Department of Fish and Wildlife Olympia Washington USA

## Abstract

Sardine (*Sardinops* spp.) are ecologically important forage fishes distributed globally across temperate, coastal upwelling zones and, when abundant, they support major fisheries. Previous genomic analyses of Pacific Sardine ( *S. sagax* ) in the Northeast Pacific detected the presence of Japanese Sardine ( *S. melanosticta* ), a species typically found in the Northwest Pacific, along the west coast of North America starting in 2022 over multiple years. To facilitate continued monitoring, we developed a highly accurate species identification Genotyping‐in‐Thousands‐by‐sequencing (GT‐seq) panel consisting of 88 single nucleotide polymorphisms (SNPs). This panel was constructed by utilizing low‐coverage, whole‐genome sequence data to identify highly divergent, genome‐wide loci between Pacific and Japanese Sardine, enabling unambiguous identification of these morphologically indistinguishable species as well as the detection of hybrids. Using the novel panel, we genotyped 1821 sardine samples from the Northeast Pacific and identified 35 hybrid individuals, which were collected from 2023 to 2025 and are the first known observation of such hybrids. Most were F1 hybrids (33); however, two individuals collected in 2025 appeared to be backcrosses with *S. sagax* . Our newly developed panel is a powerful resource for the continued monitoring of Japanese Sardine in the Eastern Pacific and is critical for investigating the potential fitness consequences of hybridization with Pacific Sardine.

## Graphical abstract

Using a newly developed nuclear Genotyping‐in‐Thousands‐by‐sequencing (GT‐seq) panel that unambiguously differentiates Japanese and Pacific Sardine, we report the first detection of *Sardinops* spp. hybrids. Previous genomic analyses of Pacific Sardine in the Northeast Pacific detected the presence of Japanese Sardine along the west coast of North America starting in 2022. Our results provide a clearer understanding of the resulting fitness consequences of hybridization in Japanese and Pacific Sardine which will help stock assessors and managers move forward on best scientific approaches in this historically important fishery.

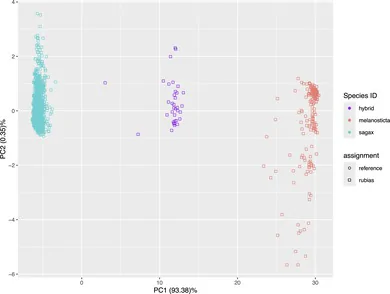

## 1 Introduction

**FIGURE 1:**
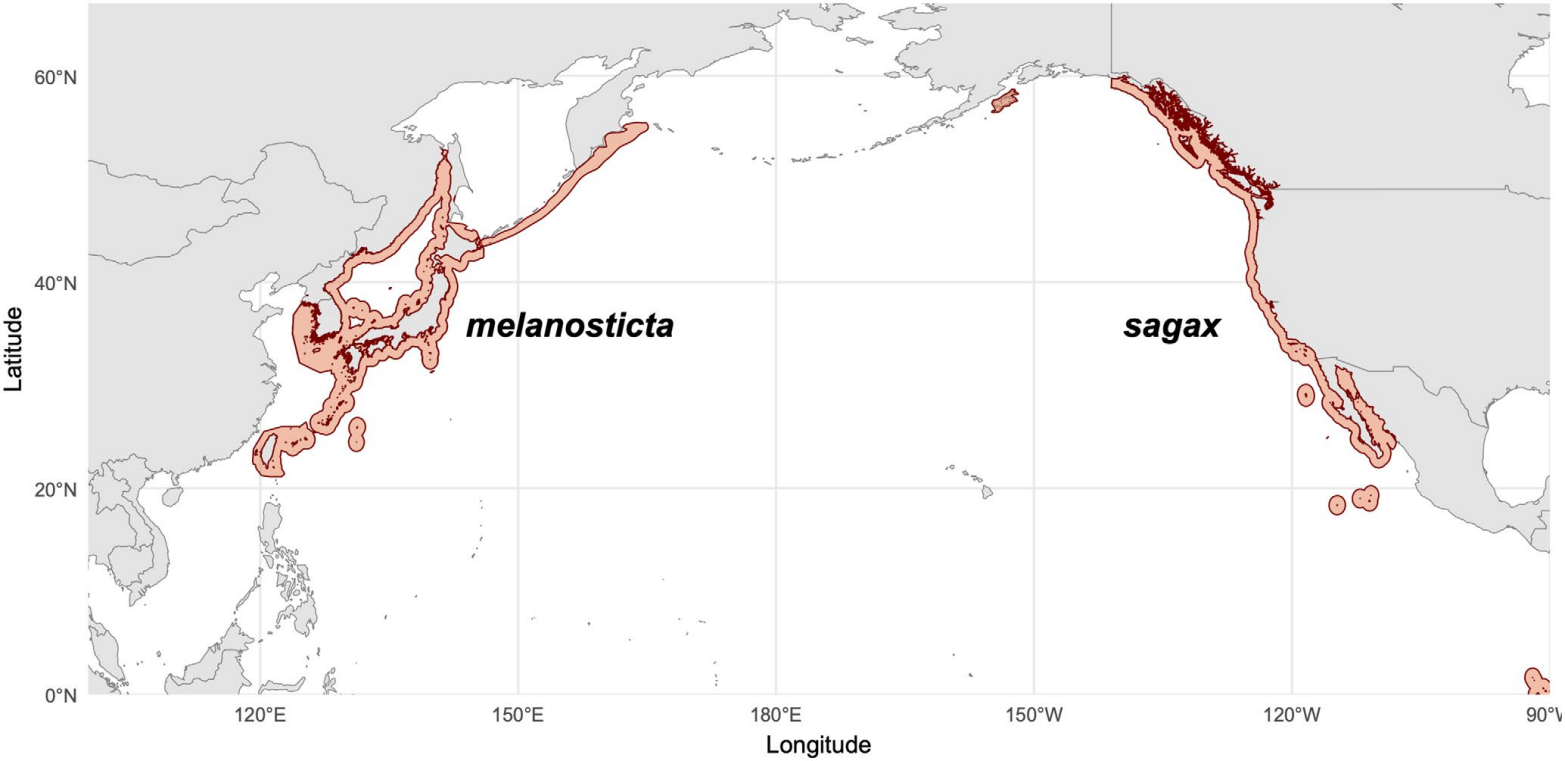
Generalized coastal distributions for the two species of sardine in the North Pacific, *Sardinops melanosticta* and *S. sagax* . Note that the Japanese and Russian fisheries routinely take *S. melanosticta* offshore to 170E and 40S.

Sardine (*Sardinops* spp.) are key ecological forage fishes in temperate, coastal upwelling zones and important fishery species when abundant. Pacific Sardine ( *Sardinops sagax* ) occur in the Eastern Pacific from Chile north to Alaska, USA. Historically, Pacific Sardine represented the most productive fishery in the Western Hemisphere and supported fisheries in Canada, the U.S., and Mexico, but recent population estimates have been low and the directed commercial fishery has been closed in U.S. waters since 2015. Japanese Sardine (*Sardinops melanostica*), which commonly occur in the Northwest Pacific from the East China Sea northward into the Sea of Okhotsk and occasionally into the Bering Sea, also undergo fluctuations in abundance characteristic of coastal pelagic species. In contrast to Pacific Sardine, recent Japanese Sardine biomass estimates have indicated a steady increase from a period of low abundance with increasing catches reported in the northern portion of their range (Furuichi et al. 2025; Yang et al. 2023). These sister taxa, which are indistinguishable based on meristic and morphometric characters (Hubbs 1929; Parrish et al. 1989), have historically exhibited allopatric distributions with cold and warm water temperature boundaries in the higher and lower latitudes of the North Pacific acting as effective dispersal barriers (Figure 1; Bowen and Grant 1997; Grant and Bowen 1998).

In the initial stages of a population genomic analysis of Pacific Sardine in the Northeast Pacific, Japanese Sardine were detected along the west coast of North America in 2022 (Longo et al. 2024). Although the mode of dispersal has not been definitively determined, Longo et al. (2024) hypothesized that recent warming trends opened a suitable habitat corridor that an expanding Japanese Sardine population followed east across the high latitudes of the North Pacific. Upon this discovery, a Genotyping‐in‐Thousands‐by‐sequencing (GTseq; Campbell et al. 2015) panel targeting three regions with interspecific fixed differences in the mitochondrial genome was developed for species identification. This panel was used to genotype samples collected during coast‐wide surveys from 2013 to 2024. Japanese Sardine have consistently been detected each year since the initial observation in 2022 but not in samples from 2013 to 2021 (Longo et al. 2024; Longo, James, et al. 2025). However, because the panel targets mtDNA which is maternally inherited and does not recombine, it lacks the capability to detect hybridization.

The ecological and evolutionary impacts of the presence of Japanese Sardine in the Eastern Pacific are important to evaluate. In particular, the potential outcomes of hybridization with Pacific Sardine have emerged as a topic of concern for management (PFMC Decision Summary Document, November 2025, https://www.pcouncil.org/november‐2025‐decision‐summary‐document/). No hybrids were detected in low coverage whole‐genome sequence (lcWGS) data; however, samples used for lcWGS were collected in 2021 and 2022, with Japanese Sardine only being detected in 2022, making the presence of hybrid offspring unlikely (Longo et al. 2024). Although these taxa are closely related with an estimated divergence time of only ~200,000–300,000 years ago (Bowen and Grant 1997), significant genomic differentiation appeared pervasive across all chromosomes, including species‐specific structural rearrangements (Longo et al. 2024). Frequently, closely related taxa can hybridize, however, fitness consequences in hybrids vary with increasing likelihood of decreased fitness (e.g., sterility or inviability) with increased genetic divergence (Burke and Arnold 2001; Coyne and Orr 2004). Understanding the fitness consequences of hybridization in this ecologically important and historically valuable fishery is critical to informed and effective management. In other systems, lcWGS data have proven to be a powerful resource for identifying divergent loci amongst populations or species of interest and developing informative nuclear GTseq panels (Beck et al. 2025; Beemelmanns et al. 2025).

Here we leveraged a lcWGS data set to identify highly differentiated loci between Pacific and Japanese Sardine and developed a nuclear GTseq panel capable of accurately differentiating the two cryptic species and identifying hybrids. We genotyped 1821 *Sardinops* samples which included 35 hybrids: 33 F1 hybrid individuals collected from 2023 to 2025 from Washington, U.S. to California, U.S., and two backcrosses with *S. sagax* collected in 2025 from Southern California, U.S. These individuals represent the first known observations of Japanese and Pacific Sardine hybrids.

## 2 Methods

### 2.1 Sample Collection

*Sardinops* tissue samples used for sequencing in this study (*n* = 2750) were collected from 2022 to 2025. Samples from 2022 to 2024 (*n* = 1705) were collected on the California Current Ecosystem Surveys (CCES), also known as the Coastal Pelagic Species (CPS) survey, conducted by the Southwest Fisheries Science Center (SWFSC) or obtained opportunistically from collaborators. In 2025, the CCES was integrated with the Joint U.S.‐Canada Integrated Ecosystem and Pacific Hake Acoustic Trawl Survey conducted by the Northwest Fisheries Science Center and is now known as the Integrated West Coast Pelagics Survey (IWCPS). On the inaugural 2025 IWCPS, 1045 *Sardinops* tissue samples were collected. The CCES/IWCPS runs from late June through late September or early October and is typically conducted aboard a NOAA fishery survey vessel using acoustics and offshore trawling, or acoustics and purse‐seining from fishing vessels in the nearshore environment (see Clemons et al. 2025; Dorval et al. 2022; Renfree et al. 2023, 2024 for a summary of methods). It is important to note that sampling effort and the spatiotemporal extent of sampling were not equivalent for each sampling year. In addition, sampling of sardine for genetic material was not synoptic over the four‐year period due to variation in sampling efforts, which were not originally designed to track the presence of Japanese Sardine. Tissue samples (caudal muscle or fin clips) were preserved at sea in 100% ethanol and archived at the SWFSC. A subsample of tissue was removed and DNA was extracted by adding 150 μL 10% Chelex resin beads suspended in deionized water and heating to 60°C for 20 min, followed by 103°C for 25 min. Most CCES samples in this study (collected from 2022 to 2024) were previously extracted (extractions were stored at −20°C) and genotyped using a mitochondrial GTseq panel (Longo et al. 2024; Longo, James, et al. 2025).

### 2.2 Age Determination

Sagittal otoliths were extracted, cleaned with water, placed in 0.6 mL microcentrifuge tubes or gel capsule, allowed to dry overnight, and assigned unique individual IDs. Whole otoliths were submerged in water or ethyl alcohol in a small dish with a black background and viewed under reflected white light using a Leica MZ10 F or M80 stereomicroscope. Otoliths were aged using white light within 3 min of submersion without knowledge of species, month of capture, sex, length, or weight (Dorval et al. 2025; Yaremko 1996).

### 2.3 Panel Development

Low coverage whole genome sequence data from Pacific Sardine ( *S. sagax* ) and Japanese Sardine ( *S. melanosticta* ) collected in the Eastern Pacific (Longo et al. 2024) were queried for highly differentiated loci to develop a species‐specific identification GTseq panel (Campbell et al. 2015). Pairwise F_ST_ estimates from comparisons between 50 Japanese Sardine and 295 Pacific Sardine (see Longo et al. 2024 for details) were used to identify strongly differentiated, informative candidate loci distributed across the Pacific Sardine genome (BioProject PRJNA1094947). Specifically, the 10 loci with the highest *F*_ST_ from each of the 24 chromosomes were targeted for primer design following Campbell et al. (2015). FASTA sequences of target SNPs with 150 bp upstream and downstream flanking regions were imported from the reference genome into Geneious v 2025.0.3. The Design New Primers tool, which utilizes Primer3 (Rozen and Skaletsky 2000), was used to design primers with the following criteria: product size between 100 and 120 bp, primer size 18–25 bp (optimal 20 bp), annealing temperature 57°C–63°C (optimal 60°C), GC 20%–80% (optimal 50%), max temperature difference between pairs 2C, and max dimer temperature of 47°C. In instances when two SNPs were in close physical proximity, an effort was made to include both in target amplicons. 179 of the 240 candidate loci were suitable for primer design.

The resulting 179 loci primer set was first evaluated using Japanese and Pacific Sardine individuals (46 Japanese and 45 Pacific Sardine) previously identified with lcWGS and mitogenome data (Longo et al. 2024), and will hereafter be referred to as the reference sample set. During panel optimization, loci that received a disproportionately large share of sequencing reads (> 10%), did not amplify well, or were non‐informative were dropped. The first round of optimization resulted in 96 primer pairs. A second and final round of optimization resulted in 84 primer pairs targeting 88 informative SNPs (4 primer pairs included two linked SNPs). The genomic distribution of the final panel loci was visualized by plotting SNP positions on the *S. sagax* reference genome using the R package karyoploteR (Gel and Serra 2017).

### 2.4 GTseq Library Preparation, Sequencing, and Species Identification

GTseq libraries were prepared using a two‐step PCR process. PCR1 reactions were prepared using Qiagen Multiplex Plus Master Mix (5.5 μL master mix, 1.5 μL pooled primer mix [10 μM of each primer], and 2 μL template DNA) or Qiagen HotStar Master Mix (5 μL master mix, 2 μL pooled primer mix, 1 μL water, and 2 μL template DNA). Following an initial denaturation at 95°C for 15 min (MultiplexPlus Mix) or 5 min (HotStar Mix), thermal cycling parameters were as follows: five rounds of 95°C for 30 s, 57°C for 90 s, 72°C 2 min, 10 rounds of 95°C for 30 s, 65°C for 90 s, 72°C 30 s, followed by an infinite hold at 4°C.

PCR2 was performed to add Illumina i5 and i7 index sequences. PCR2 reactions were prepared using Qiagen Multiplex Plus Master Mix or Qiagen HotStar Master Mix using the following ratio: 5.5 μL master mix, 2 μL i5 index at 10 μM, 2 μL i7 index primer at 10 μM, and 3 μL 1:10 diluter PCR 1 as template. Following an initial denaturation at 95°C for 15 min (MultiplexPlus Mix) or 5 min (HotStar Mix), thermal cycling parameters were as follows: ten rounds of 98°C for 10 s, 65°C for 90 s, 72°C 30 s, followed by a final extension at 72°C for 5 min and an infinite hold at 4°C.

Indexed PCR samples were cleaned and normalized using Charm Biosciences Just‐a‐Plate kit following manufacturer's protocol. Pooled, normalized PCR reactions (libraries) were size‐selected and cleaned up using a two‐sided selection process with Ampure XP magnetic beads following manufacturer's protocol. Specifically, 75 μL of AMPure XP beads were mixed with 150 μL of pooled library in a 1.5 mL lo‐bind tube. Each tube was then placed on a magnetic rack, and 200 μL of the cleared supernatant was transferred to a fresh 1.5 mL lo‐bind tube. Another 100 μL of magnetic beads were then mixed with the cleared supernatant, and each tube was placed in the magnetic rack. The supernatant was discarded, and the immobilized beads were washed twice with 70% ethanol before eluting libraries. All libraries were prepped for paired‐end (2 × 150 bp) sequencing on an Illumina MiSeq using V2 chemistry kits following Illumina protocols and loaded at a target concentration of 11 pM with 5% PhiX. 570 of the 1705 CCES samples collected in 2022–2024 were sequenced with a Nano kit and then the remaining 1135 samples with a Micro kit. The 1045 IWCPS samples collected in 2025 were sequenced using a Micro kit.

Demultiplexed FASTQ files for each individual sample were genotyped using the bioinformatic pipeline *GT‐score* (McKinney et al. 2020; https://github.com/gjmckinney/GTscore). *ProbeDesign_GTscore.Rmd* was used to generate in silico primer‐probe sequences to specifically target GTseq amplicons. Specifically, primer‐probe sets consisted of the forward locus‐specific primers of PCR1 and probes targeting desired SNPs and flanking regions. Primers targeting more than one SNP had an additional set with a second probe. *AmpliconReadCounter.pl* was used to match primer‐probe sets to each sample's amplicon sequences to count the occurrence of each unique sequence and call genotypes. Sequences that did not align were excluded as off‐target and reports were generated by individual and locus. Individuals were flagged as potentially contaminated when the percent of heterozygous loci with significantly different allele depths > 0.25 (conscore from *GT‐score*) and heterozygosity > 0.25. That is, individuals were flagged when more than 25% of loci were heterozygous and greater than 25% of those loci exhibited depth ratios significantly skewed from the expected 1:1 ratio.

Next, the R package *Rubias* v0.3.2 (Moran and Anderson 2019), which uses a Bayesian inference for genetic stock identification, was used to perform individual assignments through comparisons to GTseq genotype data from the reference sample set. The reference sample set consisted of the known, lcWGS samples of Japanese and Pacific Sardine used in the GTseq panel development. The reference sample set was tested with the *self_assign()* function, which uses a leave‐one‐out approach, to make sure *Rubias* was correctly assigning species to known individuals. For species identification, we ran *infer_mixture()* using default parameters (MCMC method, 2000 reps, and 100 burn‐in) and allele calls from the reference sample set. Individual species identification was made based on the highest posterior mean of group membership (PofZ) for the two possible species (i.e., *melanosticta* or *sagax*).

Potential hybrids were flagged using *Rubias* Z score, a standardized z‐statistic obtained from the individual's log‐likelihood. Specifically, individuals with a Z score < −10, which is considerably lower than a pure member of either parental species, were flagged as hybrids. Additionally, allele read depth ratios were visualized for all potential hybrids to further screen for potential contamination (i.e., depth ratios that skewed from the expected 1:1) using the *plotGenotypes_sample* function in *GTscore*. To visualize interspecific differentiation and potential hybridization, all samples passing quality and contamination filters were run in a PCA as implemented in the R package *adegenet* v2.1.11 (Jombart 2008) and in *STRUCTURE* v2.3.4 (Pritchard et al. 2000), a model‐based Bayesian clustering analysis. *STRUCTURE* and associated analyses were run with the R package *DartR.popgen* v1.0.0 (Gruber et al. 2018), which utilizes *StrataG* functions wrappers (Archer et al. 2017). For *STRUCTURE*, we ran three replicates for each number of genetic clusters tested (*K* = 1–5), each with a burn‐in of 50,000 iterations and 100,000 MCMC replicates with admixture allowed and no prior location information. The most likely number of clusters (*K*) across replicate runs was evaluated using the Evanno method (Evanno et al. 2005), which assesses the rate of change in log probability of the data between successive values of *K* (Δ*K*). Finally, *CLUMPP* v1.1.2 (Jakobsson and Rosenberg 2007) was used to summarize results across replicate *STRUCTURE* runs in *DartR.popgen* and final plots were created in R with *ggplot* (Wickham 2016; Wickham et al. 2019). Average individual membership coefficients (Q values) to each cluster were taken from *CLUMPP* to assess for potential backcrossing.

## 3 Results

### 3.1 Nuclear GTseq Panel Performance, Species Identification and Putative Hybrids

**FIGURE 2:**
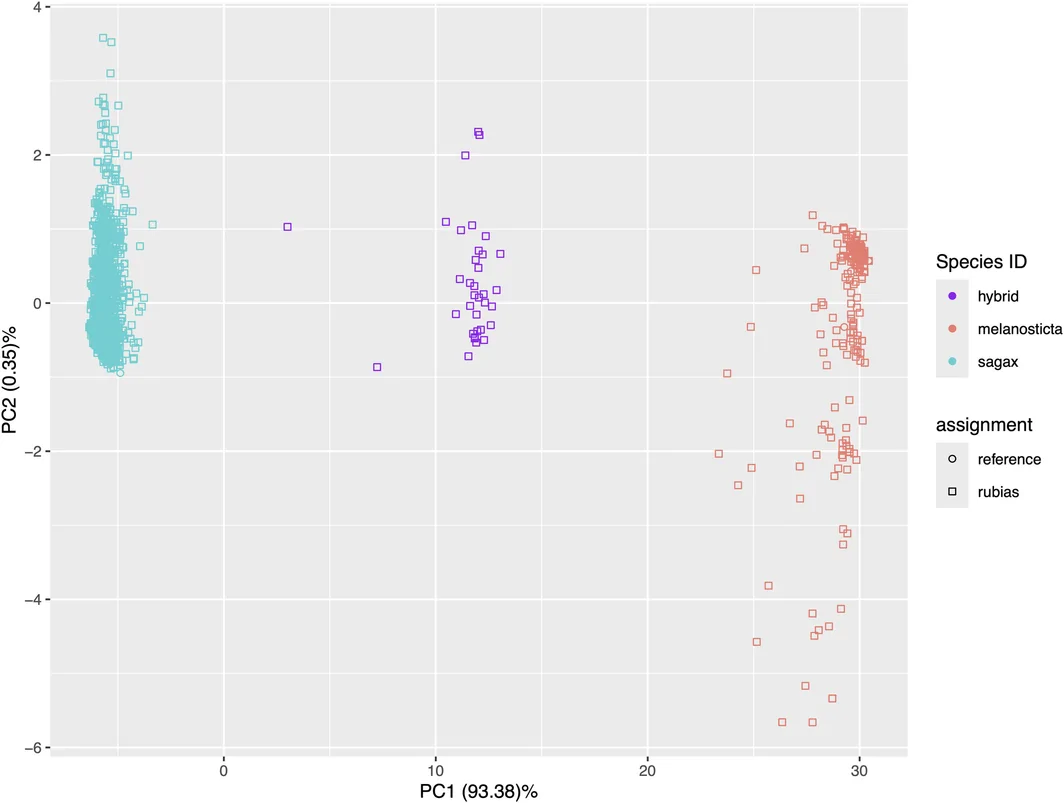
Principal component analysis on 84 loci (88 SNPs) GTseq panel from 1821 samples passing quality filter and 96 reference samples of sardine.

**FIGURE 3:**
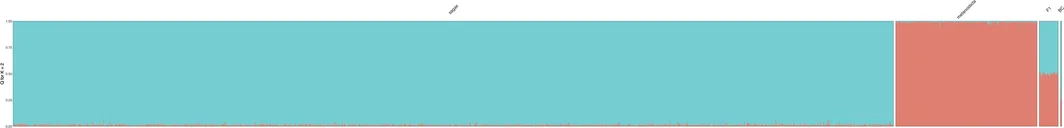
STRUCTURE results for *K* = 2 on 84 loci (88 SNPs) GTseq panel from 1821 samples of sardine passing quality filter.

88 SNPs with *F*_ST_ ≥ 0.889 (mean 0.978) from 84 primer pairs were included in the final GTseq dataset, which were widely distributed throughout the *S. sagax* reference genome (Table S1 and Figure S1). All 91 individuals in the reference sample set were assigned to the correct species with high confidence using the leave‐one‐out *self_assign()* function in *Rubias*. Overall, 2750 individual sardine samples were sequenced and 1821 passed quality and contamination filters. Most samples either genotyped well or very poorly, indicating template DNA quality may have been an issue for some samples. Twelve individuals were flagged as contaminated; eight based on the *conscore* and heterozygosity filters and four putative hybrids that showed skewed allele ratios using *plotGenotypes_sample*. In total, 247 *S. melanosticta* , 1539 *S. sagax* , and 35 hybrids were identified in *Rubias* and visualized in both the PCA (Figure 2; Figure S2 and Table 1) and *STRUCTURE* plots (Figure 3 and Figure S3). *K* = 2 was the most likely number of clusters from the Evanno method (Δ*K*; Table S2).

**TABLE 1:** *Sardinops* species counts by year.

Year	*sagax*	*melanostica*	Hybrid	Sum
2022	51 (82.3%)	11 (17.7%)	0	62
2023	205 (67.7%)	96 (31.7%)	2 (0.66%)	303
2024	453 (84.8%)	69 (12.9%)	12 (2.3%)	534
2025	830 (90.0%)	71 (7.7%)	21 (2.3%)	922
Total	1539	247	35	1821

**FIGURE 4:**
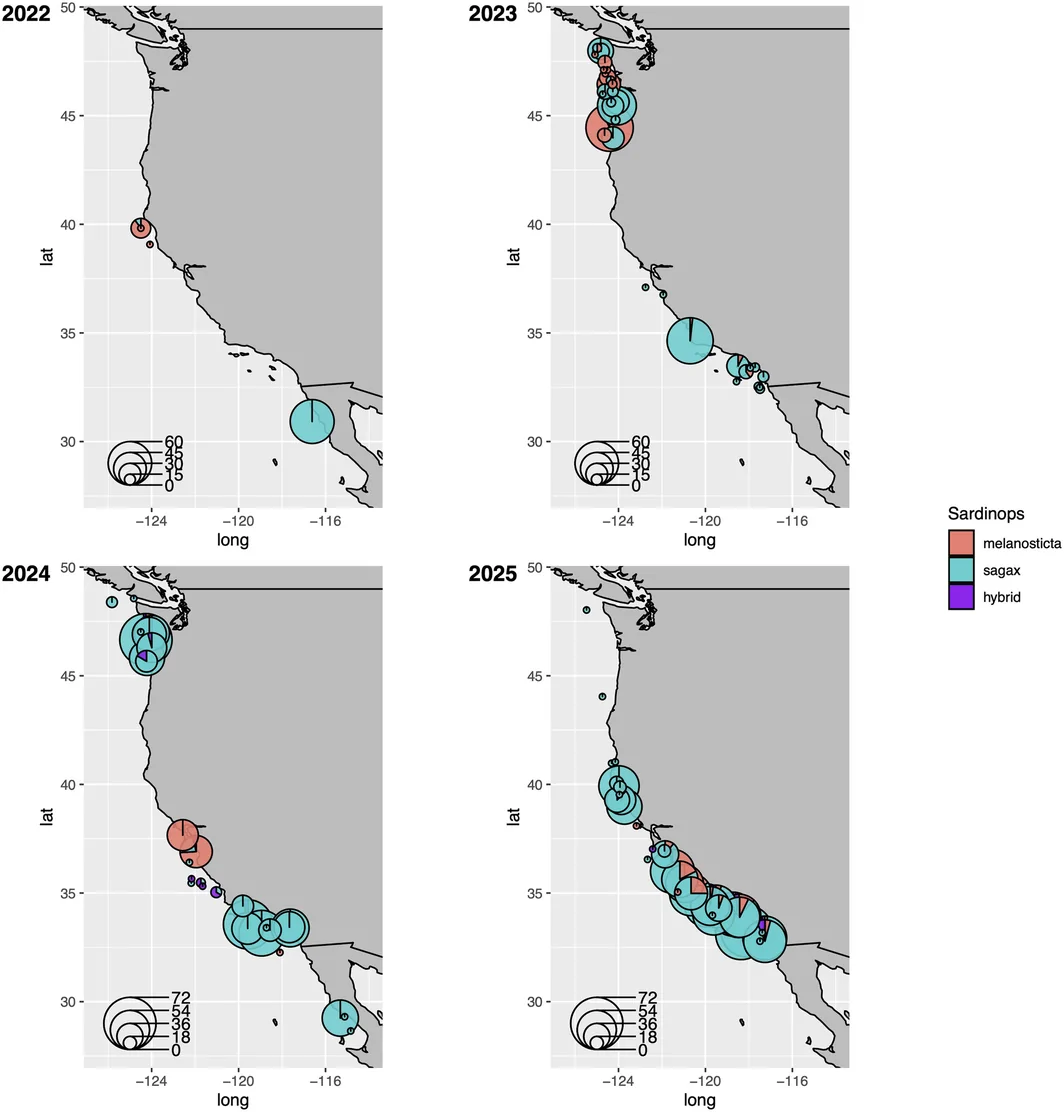
Sardine sampling locations (i.e., trawl, purse seine, or opportunistic) from 2022 to 2025. Pies represent the relative proportion of species and hybrids per sampling effort and size of pie is relative to number of individuals.

Hybrid individuals were collected in 2023 (*n* = 2), 2024 (*n* = 12), and 2025 (*n* = 21) from coastal waters off Washington south to California, U.S. (Figure 4; Table 1 and Table S3). Most of the CCES samples collected between 2022 and 2024 were previously genotyped (1490 of 1705) with a mitochondrial species identification GTseq panel (Longo et al. 2024; Longo, James, et al. 2025). Species identifications from the two panels matched in all non‐hybrid individuals. Nine hybrids had *sagax* mtDNA haplotypes, three had *melanosticta*, while the remaining 23 were not previously genotyped (Table S3). Two hybrids were identified as backcrossed to *S. sagax* based on PCA and STRUCTURE plots, and on expected ratios of heterozygous to homozygous loci. Although only 12 of the F1 hybrids had mtDNA assignments, the parental haplotype proportions (75% *sagax*, 25% *melanosticta*) were generally congruent with the observed species ratio in our data set (Table 1). Age data were available for 14 of the 35 hybrids, which ranged from age 0 to 3 (Table S3).

## 4 Discussion

Here, we leveraged the power of lcWGS data to design a species identification GTseq panel consisting of highly differentiated loci distributed across the genome and report the first observations of Japanese and Pacific Sardine hybrids. The detection of F1 and backcrossed hybrids indicates that pre‐and postzygotic barriers are permeable in these sister taxa but questions remain about potential hybridization fitness outcomes.

In addition to 33 F1 hybrids, we detected two backcrosses with *S. sagax* (F1 x *S. sagax* ; samples PS173‐2025‐021 and PS173‐2025‐033; Table S3). In the PCA, hybrids generally grouped together on PC1 between *S. sagax* and *S. melanosticta* with the backcrosses falling out closer to *S. sagax* (Figure 2). Similarly, in *STRUCTURE* plots hybrids roughly assigned equally to each parental species except for the two backcrosses, which assigned more to *S. sagax* (Figure 3 and Figure S3). Additionally, the F1 hybrids roughly met the expected percentage of 100% heterozygous loci with nearly fixed interspecific loci, as did the backcrosses with ~50% heterozygous and 50% homozygous, although these ratios can vary with recombination patterns and genomic distribution of panel loci (Table S3). We first identified hybrids in 2023, 1 year after the initial detection of Japanese Sardine in the Eastern Pacific in 2022 (Longo et al. 2024). Hybrids with age data ranged from 0 to 3. Interestingly, two individuals aged 3 were collected in 2024, indicating they were spawned in 2021 (Table S3). Therefore, Japanese Sardine were likely already present in the Eastern Pacific in 2021 and went undetected until 2022 when they were collected in relatively high abundance during the CCES (~40% of genotyped samples; Longo et al. 2024). In 2021 the nearshore portion of the CCES did not collect genetic samples, which could explain the absence of Japanese Sardine in earlier sample collections. It is possible that Japanese Sardine arrived in the Eastern Pacific in 2021 and initially remained nearshore, and therefore were not detected until 2022. As noted in the methods, sampling effort and spatiotemporal extent of sampling of sardine and sardine genetic tissue samples by the CCES/IWCPS survey were not equivalent amongst sampling years. Particularly, only a limited number of genetic tissue samples were taken prior to the detection of Japanese Sardine. Therefore, apparent differences in the relative proportion and distribution of each species amongst years (Figure 4) should be interpreted with caution as they may be sampling artifacts.

The detection of F1 and backcrossed Japanese and Pacific Sardine hybrids answers some important questions about the potential evolutionary outcome of hybridization in these cryptic, sister taxa, however questions remain about hybrid fitness. Postzygotic genetic barriers between closely related taxa often manifest in hybrids, where genetic differentiation can result in decreased fitness, inviability, or sterility due to incompatibilities of alleles that were alternatively fixed in the previously isolated taxa, also known as Bateson‐Dobzhansky‐Muller (BDM) incompatibilities (Bateson 1909; Dobzhansky 1937; Muller 1942; Coyne and Orr 2004). Later generation hybrids (e.g., backcrosses and F2 hybrids) frequently experience more serious fitness consequences than F1 hybrids (Coyne and Orr 2004). Although divergence between Japanese and Pacific Sardine occurred around 200,000–300,000 years ago (Bowen and Grant 1997), significant genome‐wide differentiation has accumulated during that time, including putative species‐specific chromosomal inversions (Longo et al. 2024). Genomic structural rearrangements such as chromosomal inversions can also result in lower hybrid fitness or sterility due to reduced recombination in heterokaryotypes, which can prevent coadaptive gene complexes from separating. Specifically, this allows for further divergence between chromosomal inversion karyotypes compared to freely recombining collinear regions of the genome and provides more opportunity for the accumulation of added incompatibilities (Faria and Navarro 2010). It is possible that some hybrid individuals with deleterious karyotype or gene complex combinations are sterile or have lower fitness than either parental species, and would therefore be selected against.

In taxa with gonochoristic sex determination, the heterogametic sex (i.e., XY, ZW chromosomes) may exhibit higher rates of hybrid inviability or sterility, which is also known as Haldane's rule (Haldane 1922; Coyne and Orr 2004). Hybrid inviability or sterility has also been observed in gonochoristic systems with monomorphic sex chromosomes (i.e., morphologically identical but with specific sex determining regions) such as in some frogs (Dufresnes et al. 2016) and teleost fishes (Crow et al. 2007; Tech 2006). Sex determination in sardine (*Sardinops* and *Sardinella*) is gonochoristic (Ganias 2014), however the specific mechanism is unknown and can vary within families and genera in fishes (Devlin and Nagahama 2002). In our data, mitochondrial genotypes were previously called in 12/35 of the hybrids with three *melanosticta* and nine *sagax* haplotypes (Table S3), indicating hybridization is bidirectional. Indeed, the observed proportions of *Sardinops* spp. mitochondrial haplotypes in hybrids are roughly congruent with the observed relative abundance of each species in the Eastern Pacific (Table 1). We observed no evidence for sex‐specific inviability in the 27 sexed hybrids based on the near equal ratio of sexes (13 females and 14 males, including the two backcrosses which were both male). The remaining samples were too small to confidently assign sex. Importantly, the observation of two backcrossed hybrids to *S. sagax* indicate that at least one sex of F1 hybrids is fertile. If sex determination in *Sardinops* is based on sex chromosomes, hybrids of the heterogametic sex (i.e., XY or ZW) could experience decreased fitness, including infertility, although this would be difficult to directly test (e.g., breeding experiments with genotyped animals).

On the other hand, it is plausible that *Sardinops* hybrids have similar or even increased fitness relative to the parental species and frequent hybrid matings, including backcrosses, would result in more hybrids—a process known as the ratchet effect (Allendorf et al. 2004). This could lead to expanded hybridization in the Eastern Pacific, where parental individuals (i.e., pure Japanese or Pacific Sardine) become less common and species‐specific alleles become randomly distributed across hybrid individuals. This scenario has been observed in congeneric fishes such as Rainbow and Cutthroat Trout (*Onchorynkiss* spp.; Allendorf et al. 2004), Blacktail and Red Shiner (*Cyprinella* spp.; Walters et al. 2008), and Alewife and Blueback Herring (*Alosa* spp.; Hasselman et al. 2014). Although we only have a few years of data on hybrid presence, it does not appear that hybrid frequency is increasing in our limited observations. Previous work using lcWGS data suggested low levels of nuclear introgression (mean = 0.3%) from Japanese Sardine into Pacific Sardine with two cases of mitochondrial introgression (Pacific Sardine nuclear DNA with Japanese Sardine mtDNA; Longo et al. 2024; Longo, D'Amelio, et al. 2025). These data point to past instances of hybridization and introgression, suggesting these dispersal events may be episodic in nature. However, current warming trajectories suggest that a favorable habitat corridor across the higher latitudes of the North Pacific may persist more consistently, which could result in increased connectivity between Japanese and Pacific Sardine.

The detection of hybridization, introgression, and large structural variants in *Sardinops* spp. described here and previously (Longo et al. 2024; Longo, D'Amelio, et al. 2025) has also been observed in other Clupeiforms. In Atlantic Sardine *(Sardina pilchardus)*, researchers used genomic data to identify several large inversions that covered more than half the genome, with some karyotypes correlating with locally adapted life history strategies (Sabatino et al. 2025). Similarly, researchers found that ecotype evolution in European Anchovy ( *Engraulis encrasicolus* ) was likely driven by a combination of local adaptation and admixture between geographically isolated lineages that share structural variants (Meyer et al. 2025). Both Atlantic Herring (*Clupea clupea*) and Pacific Herring ( *C. pallasi* ) have been shown to have structural variants associated with spawning timing (Han et al. 2020; Petrou et al. 2021). In migratory European shad species, the extent of hybridization between *Alosa alosa and A.* *fallax* was revealed through genetic markers, although more work is needed to understand potential fitness consequences (Antognazza et al. 2022). Collectively, these studies shed light on the major role structural variants (e.g., inversions), hybridization, and introgression have played in the evolution of Clupeiformes. Importantly, it has also clarified the population genetic structure for many of these ecologically and economically important species providing critical information on stock structure to better inform fisheries management.

The detection of Japanese and Pacific Sardine hybrids in the Eastern Pacific furthers our understanding of the potential evolutionary outcomes of this trans‐Pacific dispersal event. The observation of hybrids, particularly backcrosses, indicates the species barrier is permeable, which is congruent with signals of past introgression from previous lcWGS data. Questions remain about hybrid fitness compared to parental species as fitness consequences can manifest in later generation hybrids. Continued genetic monitoring of *Sardinops* for changing proportions of hybrid individuals, including hybrid sex ratios, will shed additional light on the nature of hybridization and introgression between these species. Additionally, generating high density SNP data (e.g., lcWGS) for F1 and backcrossed individuals could provide important insight into how species‐specific inversions may be affecting recombination. Importantly, a clearer understanding of resulting fitness consequences of hybridization in Japanese and Pacific Sardine will help stock assessors and managers move forward on best scientific approaches.

## Author Contributions

**Gary C. Longo:** conceptualization (lead), data curation (lead), formal analysis (lead), investigation (lead), methodology (lead), project administration (supporting), resources (supporting), software (lead), supervision (equal), visualization (lead), writing – original draft (lead), writing – review and editing (equal). **Flora Coden:** investigation (equal), writing – review and editing (equal). **Miasara Andrew:** investigation (supporting), writing – review and editing (supporting). **Kelsey James:** data curation (supporting), formal analysis (supporting), investigation (supporting), methodology (supporting), writing – review and editing (supporting). **Kristen Hinton:** formal analysis (supporting), investigation (supporting), resources (supporting), supervision (supporting), writing – review and editing (supporting). **Jennifer Topping:** formal analysis (supporting), investigation (supporting), resources (supporting), writing – review and editing (supporting). **Matthew Craig:** conceptualization (supporting), formal analysis (supporting), investigation (supporting), methodology (supporting), project administration (lead), resources (lead), supervision (equal), writing – original draft (supporting), writing – review and editing (supporting).

## Conflicts of Interest

The authors declare no conflicts of interest.

## Supporting information


**Figure S1:** Final GTseq panel loci (blue arrows) locations on the 24 chromosomes of the *Sardinops sagax* reference genome (length in megabases below each chromosome). Panel loci with two SNPs are circled in red. Turquoise blocks represent putative species‐specific structural variants detected in *S. sagax* .

**Figure S2:** Principal component analysis on 84 loci (88 SNPs) GTseq panel from 1821 samples passing quality filter and 96 reference samples of sardine. Individuals are color coded by number of missing loci.

**Figure S3:** STRUCTURE results for *K* = 2 on 84 loci (88 SNPs) GTseq panel from 35 Japanese and Pacific Sardine hybrid samples passing quality filter and 96 reference samples of sardine.

**Table S1:** GTseq panel loci and SNPs information. Marker name, chromosome number, position on chromosome (midpos), pairwise *F*_ST_ based on lcWGS data, Locus_ID, alleles, forward primer and reverse primer sequence. *Note:* four markers target two SNPs.

**Table S2:** Evanno table output for STRUCTURE runs. The number of clusters (k), the number of replicate runs (reps), mean log likelihood (mean.ln.k), standard deviation of log‐likelihood (sd.ln.k), first order rate of change of the likelihood (ln.pk), second order rate of change of the likelihood (ln.ppk), and ΔK (delta.k).

**Table S3:** Metadata for 35 Japanese and Pacific Sardine hybrids.

## Data Availability

lcWGS sequencing data used to identify highly divergent loci for development of the GTseq panel are deposited at the National Center for Biotechnology Information and are accessible under BioProject PRJNA1094947. GTseq panel loci primer sequences and SNP information are in Table S1. Fastq files for all samples passing QF are deposited in the Dryad repository https://doi.org/10.5061/dryad.6djh9w1hn.
